# Methicillin-Resistant *Staphylococcus aureus* Nasal Colonization among Adult Patients Visiting Emergency Department in a Medical Center in Taiwan

**DOI:** 10.1371/journal.pone.0018620

**Published:** 2011-06-13

**Authors:** Sheng-Yun Lu, Fang-Yu Chang, Ching-Chung Cheng, Keong-Diong Lee, Yhu-Chering Huang

**Affiliations:** 1 College of Medicine, Chang Gung University, Kweishan, Taoyuan, Taiwan; 2 Department of Emergency Medicine, Chang Gung Memorial Hospital at Linko, Kweishan, Taoyuan, Taiwan; 3 Department of Pediatrics, Chang Gung Memorial Hospital at Linko, Kweishan, Taoyuan, Taiwan; University of Hong Kong, Hong Kong

## Abstract

**Background:**

Within the past 10 years, methicillin-resistant *Staphylococcus aureus* (MRSA) has not only been a hospital pathogen but also a community pathogen. To understand the carriage rate of methicillin-resistant *Staphylococcus aureus* (MRSA) among the adult patients visiting emergency department (ED), we conducted this study.

**Methodology/Principal Findings:**

From May 21 to August 12, 2009, a total of 502 adult patients visiting emergency department (ED) of a tertiary care hospital in northern Taiwan were recruited in this study and surveyed for nasal carriage of MRSA. A questionnaire regarding the risk factors for MRSA acquisition was also obtained.

The overall prevalence of MRSA nasal carriage among the patients was 3.8%. The carriage rate was significantly higher in patients with risk factors for MRSA acquisition (5.94%) than those without risk factors (2.12%). Patients with urinary complaints, diabetes mellitus, chronic kidney disease and current percutaneous tube usage were significantly associated with MRSA colonization. By multiple logistic regression analysis, only current usage of catheters or tubes was the independent predictor for MRSA nasal colonization. Of the 19 MRSA, most isolates belonged to one of two linages, characterized as sequence type (ST) 239 (32%) and ST 59 (58%). The latter linage, accounting for 83% of 6 isolates from patients without risk factors, is a community-associated (CA) clone in Taiwan, while the former linage is among healthcare-associated clones.

**Conclusion/Significance:**

A substantial proportion of patients visiting ED, particularly with current usage of percutaneous catheter or tubes, in northern Taiwan carried MRSA, mostly community strains, in nares.

## Introduction

*Staphylococcus aureus* is an important pathogen in humans and causes a broad spectrum of diseases, ranging from skin and soft tissue infection, myositis, bone/joint infection, pneumonia, endocarditis, bacteremia, to life-threatening infections of septicemia, necrotizing fasciitis, and toxic shock syndrome [1]. It is always a challenge to treat infections due to *S. aureus*, particularly isolates resistant to methicillin (methicillin-resistant *S. aureus*, MRSA) and related beta-lactams. Nowadays, MRSA is endemic in most hospitals in the world and accounts for 40–60% of all nosocomial *S. aureus* infections. MRSA was usually viewed as a cause of nosocomial infection [2]. However, within the past 10 years, it has become an increasing threat to not only hospitals but also community settings [3], [4].

Community-associated MRSA (CA-MRSA) isolates established infections in patients without traditional MRSA risk factors [5]–[8] and shared common molecular characteristics which are different from healthcare-associated MRSA (HA-MRSA) isolates [6]–[9]. However, CA-MRSA clones varied in different continents, countries and even areas. CA-MRSA strains are now endemic in many US hospitals [5], [7], and about two-thirds of severe HA-MRSA infections were community-onset [10]. It is likely that these patients returned hospital settings through emergency department (ED).

In Taiwan, MRSA was first documented in early 1980s and rapidly increased in 1990s, accounting for 53–83% of all *S. aureus* isolates in most hospitals of Taiwan in 2000s [11]. In addition, CA-MRSA infections have been increasingly reported in pediatric patients since 2000 [12], [13].

Colonization of *Staphylococcus aureus* strains may serve as endogenous reservoirs for subsequent clinical infections [14], [15], and the risk was even higher for MRSA. Since patients visiting the emergency department (ED) may come from different settings, we aimed to determine in this study the prevalence of *Staphylococcus aureus* and MRSA nasal colonization among the patients visiting ED, and further to identify the risk factors for acquisition and microbiologic characteristics of MRSA. Such information can provide the extent of MRSA in ED, thus shaping strategies for prevention and treatment of MRSA, both in the hospital and community.

## Materials and Methods

Chang Gung Memorial Hospital is a university-affiliated 3000-bed tertiary teaching hospital situated in northern Taiwan. It provides primary care, secondary care, and tertiary care. Approximately 15000 patients visited the ED each month. This study was approved by the Institutional Review Board of Chang Gung Memorial Hospital. From May 21 to August 12, 2009, patients aged above 18 years old visiting ED of Chang Gung Memorial Hospital were invited and surveyed for nasal carriage of MRSA after a written consent was obtained. A questionnaire regarding the risk factors for MRSA acquisition was also obtained.

### Laboratory methods

Nasal swab samples were collected with sterile swabs from both anterior nares, then placed in the transport medium (Venturi Transystem, Copan Innovation Ltd., Limmerick, Ireland) and sent to microbiological laboratory for culture. Swabs were plated by streak plate method on Blood Agar Plate Isolates of *S. aureus* and MRSA identification by oxacillin susceptibility with the disc diffusion methods were confirmed according to the recommendations of Clinical and Laboratory Standards Institute [16].

### Antimicrobial susceptibility testing

The susceptibility of MRSA isolates to 9 antibiotics including doxycyclin, vancomycin, teicoplanin, penicillin, trimethoprim/sulfamethoxazole, erythromycin, chloramphenicol, linezolid, and fusidic acid was determined using the disk-diffusion method according to the recommendations of Clinical and Laboratory Standards Institute [16].

### Molecular typing

Chromosomal DNAs were extracted from MRSA isolates for molecular characterization. Pulsed-field gel electrophoresis (PFGE) was used to fingerprint all MRSA isolates according to the procedure described previously [13], [17]. Staphylococcal chromosome cassette *mec* (SCC*mec*) type, and the presence of Panton-Valentine leukocidin (PVL) genes were determined by PCR assays according to the procedure described previously [13], [17], [18]. Multilocus sequence typing (MLST) was performed for selective strains of representative PFGE patterns as described elsewhere [19].

### Questionnaire and Statistical analysis

Each participant, with or without the assist of their family, was requested to complete a questionnaire regarding risk factors for MRSA colonization. Demographic and clinical data were collected. Demographic data included age, gender, education level, social economic status, and smoking habits. High social economic level was defined by having both high school diploma and/or monthly salary exceeding NT 50000. Those who do not fulfill either conditions were classified as low social economic level. Clinical information regarding chief complaint for this visit to ED, recent hospitalization or outpatient department visit, dialysis, current usage of tubes (nasogastric tube, urine catheter, tracheostomy tube, drainage tube, port-A, and dialysis tube), chronic underlying disease, and recent antibiotic use within one year of enrollment were obtained. The details of their recent hospitalization history, laboratory tests, and antibiotic use were further obtained by medical chart review.

Patients with a history of hospitalization, surgery, dialysis, or residence in a long-term care facility within 1 year of enrollment, a permanent indwelling catheter or percutaneous medical device (eg, tracheostomy tube, gastrostomy tube, or Foley catheter), or a known positive culture for MRSA prior to the study [6] were classified into the group with risk factors for MRSA acquisition. Those without any of the above factors were the group without risk factors.

### Statistics

The categorical data was examined by chi-square test or logistic regression model using SPSS 16.0 statistical software. A *p* value<0.05 indicated a significant difference statistically. Risk factors associated with MRSA colonization with a *p* value<0.05 were subsequently included for further multivariate logistic regression model.

## Results

A total of 502 adult patients were enrolled in this study. 268 patients (53%) were male. The majority of the patients were over 60 years of age and the age distribution was 55(11%) patients between 19–29 years, 212 (42%) between 30–59 years and 235 (47%) over 60 years. 219 patients (44%) were classified into the group with risk factors and 283 patients the group without risk factors.

The overall prevalence of methicillin sensitive *S. aureus* and MRSA nasal carriage were 13.5% and 3.8%, respectively. The overall carriage rate of *S. aureus* was 17.3%. The carriage rate of *S. aureus* and MRSA, respectively, among the patients with risk factors were significantly higher than that of those without risk factors (p = 0.033, respectively) (Table 1).

**Table 1:** Comparison of nasal *Staphylococcus aureus* and methicillin-resistant *S. aureus* (MRSA) colonization rate between adult patients with and without risk factors.

Colonized subjects	No. (%) of subjects			Odds Ratio [95% confidence interval]	*p* value
	With risk factor	Without risk factor	Total		
Subject No.	219	283	502		
*S. aureus*	47(21.4)	40(14.1)	87 (17.3)	1.667(1.047–2.653)	0.033
MRSA	13 (5.9)	6 (2.1)	19 (3.8)	2.924(1.093–7.822)	0.033

The association of demographic and clinical factors with MRSA colonization is shown in Table 2. Univariate analysis revealed that diabetes mellitus, chronic kidney disease, current usage of percutaneous catheters or tubes, and urinary complaint for this visit were significant risk factors for MRSA colonization. After multivariate logistic regression analysis, only current usage of catheters or tubes (*p* = 0.025) was the independent predictor for MRSA colonization (Table 3). Patients more than 60 years of age and those with the education level below elementary school had a trend toward MRSA colonization. The patients not hospitalized within one year had a trend against MRSA colonization.

**Table 2:** Association of methicillin-resistant *S. aureus* (MRSA) colonization with demographic and clinical characteristics of patients visiting emergency department.

Demographic and clinical data	No. (%) of subjects		Odds ratio	95% confidence interval	*p* value^a^
	MRSA(n = 19)	Non-MRSA(n = 483)			
**Male**	7(36.8)	261(54.0)	0.496	0.192–1.282	0.141
**Age**					
19–29	0(0)	55(11.3)	1	0.939–0.976	0.997
30–59	6(31.5)	206(42.6)	0.621	0.232–1.660	0.342
> = 60	13(68.4)	222(45.9)	2.547	0.952–6.813	0.062
**Education level**					
Elementary school	13(68.4)	221(45.7)	2.569	0.96–6.87	0.06
Junior and high school	4(21.0)	186(38.5)	0.426	0.139–1.303	0.124
Colleagues	2(10.5)	76(15.7)	0.63	0.143–2.783	0.539
**Low social economic status** b	17(89.4)	419(86.7)	1.298	0.293–5.753	0.73
**Smoking habit**					
non-smoker	13(68.4)	281(58.1)	1.558	0.582–4.167	0.378
ex-smoker	4(21.0)	110(22.7)	0.904	0.294–2.780	0.861
current smoker	2(10.5)	92(19.0)	0.5	0.114–2.202	0.36
**Underlying diseases**					
DM	9(47.3)	115(23.8)	3.174	1.231–8.186	0.017*
Heart disease	4(21.0)	99(20.4)	1.102	0.355–3.423	0.866
Hypertension	10(52.6)	190(39.3)	1.914	0.742–4.938	0.179
CVD	3(15.7)	65(13.4)	1.28	0.361–4.544	0.703
Liver disease	2(10.5)	77(15.9)	0.656	0.148–2.910	0.576
Biliary system disease	0(0)	13(2.6)	1	0.946–0.98	0.48
Asthma	1(5.2)	30(6.2)	0.884	0.114–6.872	0.906
COPD	0(0)	13(2.6)	1	0.946–0.98	0.48
Bronchiectasis	0(0)	7(1.4)	1	0.947–0.98	0.606
Cancer	6(31.5)	102(21.1)	1.858	0.681–5.071	0.22
Allergic rhinitis	2(10.5)	36(7.4)	1.545	0.342–6.985	0.569
CKD	5(26.3)	48(9.9)	3.47	1.186–10.152	0.023*
Chronic seizure disease	1(5.2)	10(2.0)	2.771	0.335–22.894	0.324
Autoimmune disease	0(0)	8(1.6)	1	0.945–0.979	0.572
TB	0(0)	17(3.5)	1	0.940–0.978	0.395
**Other risk factors**					
Duration of previous hospitalization within 1 year					
Never	8(42.1)	292(60.4)	0.418	(0.165–1.061)	0.066
<7 days	5(26.3)	68(14.0)	2.059	(0.718–5.902)	0.17
>7 days	6(31.5)	99(20.4)	1.683	(0.624–4.541)	0.299
Culture during last hospitalization^c^					
No	15(78.9)	390(80.7)	0.692	0.223–2.146	0.524
Other bacteria	3(15.7)	62(12.8)	1.21	0.343–4.272	0.767
*S. aureus*	1(5.2)	10(2.0)	2.511	0.305–20.69	0.376
Current usage of catheters or tubes^d^	7(36.8)	59(12.2)	4.192	1.587–11.071	0.002*
Current usage of NG tubes	1(5.3)	1(0.2)	1.733	0.217–13.850	0.599
Current antibiotics use	7(36.8)	165(34.1)	1.124	0.434–2.91	0.809
Antibiotics within a year	12(63.1)	211(43.6)	2.21	0.855–5.710	0.102
Dialysis	2(10.5)	17(3.5)	0.116	0.689–15.088	0.116
**Chief complaint** e					
Respiratory	1(5.2)	78(16.1)	0.288	0.038–2.192	0.23
Gastrointestinal	5(26.3)	137(28.3)	0.902	0.319–2.552	0.846
Urinary	4(21.0)	32(6.6)	3.758	1.178–11.986	0.017*
skin superficial infection	1(5.2)	26(5.3)	0.976	0.125–7.601	0.982
other systemic symptoms	8(42.1)	210(43.4)	0.945	0.374–2.392	0.906

a Fisher's exact test instead of Pearson's chi-square test was performed when any expected count was less than 5 by statistical analysis.

b Low social economic status was defines as patients other than those with education level above senior high and a monthly income more than 50,000 NT.

c Including wound or surgical site culture, blood culture, sputum culture, and urine culture.

d Including Foley, port A, percutaneous drainage tubes, and catheter for dialysis.

e The chief complaints were classified according to the systems of the body affected.

**Table 3:** Comparison of nasal *Staphylococcus aureus* and methicillin-resistant *S. aureus* (MRSA) colonization rate between subjects using catheters/tubes or not.

Carrier	No.(%) of subjects			Odds Ratio[95% confidence interval]	p value
	subjects using catheters or tubes	subjects not using catheters or tubes	Total		
Subject No.	66	436	502		
MRSA	7(10.6)	12(2.8)	19(3.8)	4.192(1.587–11.071)	0.002
*S. aureus*	21(31.8)	66(15.1)	87(17.3)	2.616(1.464–4.674)	0.001

The molecular characterization of all 19 MRSA isolates is shown in Table 4. A total of six PFGE were identified with three major patterns (type A, 21%; type C, 32% and type D, 26%). Most MRSA isolates belonged to two lineages as sequence type (ST) 59 and ST 239. ST 59 linage was further classified into two clones, characterized as PFGE C/SCC*mec* IV/PVL-negative and PFGE D/SCC*mec* V_T_/PVL-positive. These two clones accounted for 83% of 6 isolates from patients without risk factors, and are community-associated (CA) clones in Taiwan.

**Table 4:** Distribution of PFGE pattern and other molecular characteristics of 19 methicillin-resistant *Staphylococcus aureus*, stratified by with or without risk factors.

Characteristics	No.(%)of isolates	A	B	C	D	F	BM
Without risk factors	6 (31.5)	1 (16.6)	0	2 (33.3)	3 (50)	0	0
With risk factors	13 (68.4)	3 (23.0)	2 (15.3)	4 (30.7)	2 (15.3)	1(7.6)	1(7.6)
Total	19	4	2	6	5	1	1
MLST types		239	239	59	59	5	45
SCC*mec* type		III*	III*	IV	V_T_	II	UT
PVL genes-positive		0	0	0	5	0	0

*included its variants IIIa and IIIb.

MLST, multilocus sequence type; SCC*mec*, staphylococcal chromosome cassette type; PVL, Panton-Valentine leukocidin; UT, untypeable.

All 19 MRSA isolates were susceptible to vancomycin, linezoid, teicoplanin, and fusidic acid. Susceptibility to trimethoprim-sulfamethoxazoe (TMP-SMX), clindamycin, doxycyclin, and erythromycin was detected in 73.7%, 21.0%, 68.4%, and 21.0% of the isolates, respectively. For the 6 isolates from patients without risk factors, the susceptibilities to clindamycin, doxycyclin, and erythromycin were significantly higher than those of the isolates from patients with risk factors.(P = 0.041, P = 0.05, P = 0.041, respectively) (Table 5).

**Table 5:** Antimicrobial susceptibility of 19 methicillin-resistant *S. aureus* isolates, stratified by patients with or without risk factors.

Antibiotics	Without risk factors(n = 6)	With risk factors(n = 13)	P value
Vancomycin	6 (100%)	13 (100%)	
Linezoid	6 (100%)	13(100%)	
Teicoplanin	6 (100%)	13(100%)	
Fusidic acid	6 (100%)	13(100%)	
Oxacillin	0	0	
Penicillin	1 (17%)	0	0.141
TMP-SMX^a^	6 (100%)	8 (62%)	0.085
Clinidamycin	3 (50%)	1 (8%)	0.041
Doxycyclin	6 (100%)	7 (54%)	0.05
Erythromycin	3 (50%)	1 (8%)	0.041

a TMP-SMX, trimethoprim-sulfamethoxazole.

P value by chi square test for the significant difference in drug resistance among colonized subjects with and without risk factors.

## Discussion

Results from this study indicated that the nasal carriage rate of MRSA among the adult patients visiting the ED of a medical center in northern Taiwan was 3.78%, a rate significantly higher than that among patients admitted to hospitals in the Netherlands (0.03% during 1999–2000) [20] and that among general population in the US (1.5% during 2003–2004) [21]. Compared with previous reports from Taiwan, the rate was significantly lower than that for adult patients hospitalized in ICUs (32%) [22] but similar to that for adults in the community and adults for health examination [23], [24]. Patients visiting emergency department may come from both community settings and healthcare facilities. Therefore, the expected nasal MRSA carriage rate among these patients should be higher than that among the community subjects, but it is not the case in the present study. The carriage rate was nearly same for both populations. Since it was harder to get access to patients with severe medical condition, we might collect samples from subjects that held characters more close to those in the community settings. However, the carriage rate was significantly higher in the patients with risk factors (5.94%) than those without risk factors (2.12%) (Table 1). Further studies are needed to confirm this finding, since nasal MRSA carriage was only identified in 19 individuals in the present study.

In the current study, 58% of MRSA isolates belonged to ST 59 linage, which included several clones with two major clones (PFGE C/SCC*mec* IV/PVL-negative and PFGE D/SCC*mec* V_T_/PVL-positive) and were community strains in Taiwan [13], [25], and the rate was even up to 83% of the isolates from those without risk factors for MRSA acquisition. In contrast, about 30% of MRSA isolates belonged to ST 239 linage, which was among worldwide epidemic clones as well as healthcare associated clones in Taiwan [17], [25]. Likewise, the isolates from the patients with risk factors were resistant to more antibiotics than those from patients without risk factors. These results were compatible with our assumption that patients visiting emergency department may come from both community settings and healthcare facilities. The issue whether CA-MRSA strain will become widespread, even in health-care facilities, in Taiwan needs further observation [26], [27].

In the current study usage of percutaneous catheters or tubes was identified as the only independent predictor for MRSA colonization. Whether biofilm-forming capacity of *S. aureus* on various indwelling devices assists its persistence in the host needs further studies. Wang et al found that smoking was a protective factor against MRSA colonization in the community setting [24], which was not confirmed in this study.

In conclusion, 3.78% of patients visiting the ED of a medical center in northern Taiwan in 2009 harbored MRSA in nares. Usage of percutaneous catheters or tubes was significantly associated with MRSA colonization. Most of the isolates belonged to ST59 linage, a community clone in Taiwan. Nasal MRSA colonization among patients visiting ED may accelerate the spread of MRSA both in the community and healthcare-associated settings.

### Perspectives

Colonization of MRSA, compared with MSSA colonization, was 4-fold more likely to develop invasive infection [15]. To avoid invasive infection among the colonized ones, prevention strategies have to be made. Several studies have shown that elimination of nasal carriage reduces the incidence of *Staphylococcus aureus*, and the carriage from other body sites usually disappeared after the nasal carriage has been treated [28]–[30]. Intervention strategies included decolonization with topical treatment (eg. mupirocin ointment to eradicate nasal carriage, tea tree oil and chlorhexidine gluconate to eradicate cutaneous carriage), oral probiotic preparation containing lactobacillus and occasional systemic antimicrobial agents [31], [32]. Nevertheless, widespread use of antibacterial agents may elicit the development of resistance. Since the relative risk of developing invasive infection after carriage is linked to the clinical comorbidities [15], carriage eradication is necessary in the hospitalized patients, especially those at high risk of MRSA acquisition as mentioned in the present study. Recent study has shown that nasal wash with water and use of nasal sprays was associated with significant decrease of *Staphylococcus aureus* colonization rate [33]. However, its clinical practicality requires further investigation.
